# Reducing endophthalmitis in India: An example of the importance of critical appraisal

**DOI:** 10.4103/0301-4738.71702

**Published:** 2010

**Authors:** Ravi Thomas

**Affiliations:** Queensland Eye Institute, University of Queensland, South Brisbane, Australia

Dear Editor,

The peer review process strives to ensure accuracy of published research. However, passing muster with reviewers (or the editorial board) does not in itself legitimize an article’s conclusions; ultimately it is the reader’s responsibility to scrutinize the data and use other available information before adopting recommendations. This is especially true of research that (rightly) questions existing dogma. Such investigations have the capability of initiating much needed cost-effective changes, but, unfortunately, also carry the potential for dire consequences. A case in point is an article pertaining to endophthalmitis in India that was recently published in an overseas journal.

The occurrence of post-cataract endophthalmitis in India has been reported to be 0.6%.[1] The publication from an Aravind Eye Care System hospital reports an incidence of 0.09% (0.02% in paying patients) and attributes this impressive reduction to their unconventional surgical protocols.[2] If the article’s results are valid, the described protocols should be considered for widespread, indeed worldwide application, however unusual, unconventional or controversial they may seem.

The system of critical appraisal that I have used and taught in journal clubs was devised by Riegelmann, supplemented by other texts.[3–5] Riegleman named his format MAARIE, each letter standing for the headings under which the article is analyzed: Methods, Assignment, Assessment, Results, Interpretation, Analysis and Extrapolation. We ask several questions under each heading. As an Indian I prefer to substitute “Study” for “Methods” and teach critical appraisal using the SAARIE format.

It is impossible to provide a full critique of the article in this brief communication, but in order to illustrate the process I will apply the relevant parts of SAARIE and incorporate important background knowledge of cataract surgery in India to re-analyze the data and conclusions.

One of the questions asked under the heading Study, (S in SAARIE), is whether the study design was appropriate for the question being asked. We know that incidence should be determined by a prospective study, not a retrospective one. Incidence calculated from a retrospective study should be viewed with caution.

Assignment, the first A in SAARIE also highlights that cases were obtained retrospectively. Under this heading we also look for confounding. A confounder has several attributes: It is a factor extraneous to the question being asked that can affect the outcome, is different between the analyzed groups and is not in the causal pathway. “Charity” patients are extraneous to the question being asked but, as we will see, they can affect the outcome and are unequally distributed between the analyzed groups. We must consider “charity” a potential confounding factor.

Equally important is the Assessment of outcomes (the second A in SAARIE). The selected outcome measure must be appropriate, accurate, precise and should be evaluated in a masked manner with adequate follow-up. The detection of endophthalmitis is certainly an appropriate outcome measure but the article did not adequately define endophthalmitis or the details of how it was diagnosed; nor did it provide data for the accuracy and precision of diagnosis. The “clinical impression” of doctors at various levels of training rather than a precise definition and a clear description of methods used for the diagnosis is not acceptable. We also need to keep in mind that some of the diagnoses were made in the environs of cataract screening camps in remote areas; anyone who has attended such camps knows the attendant diagnostic limitations. Add to this the fact that the commonest organism grown was *Nocardia*, quite different (and clinically severe) from the spectrum reported from other hospitals, and we begin to suspect that the commoner organisms and early cases were likely missed.

While on outcome measures, we also look at completeness of follow-up and build this into our analysis. It is a little difficult to reconcile the follow-up periods with the article submission date, but we are more concerned about the loss to follow-up. The loss to follow-up was 6% amongst the paying patients and 16% in the “charity” group. While this does not seem too high, in the presence of a low rate of the event in question, such a loss to follow-up can change the results considerably and deny robust conclusions.[5] One acceptable way to deal with loss to follow-up is to analyze the worst case scenarios: we assume that all patients lost to follow-up developed endophthalmitis. If this worst case endophthalmitis rate was still acceptably low (and there were no other fatal flaws), we could still be convinced to change our practice. Overall 5,586 patients were lost to follow-up. The worst case scenario is an overall endophthalmitis rate of 13%, 6% for paying patients and 16% for charity patients.

The informed reader would realize at this point that given the fatal flaws in study design and assessment of the outcome, the results cannot be applied. I will however continue the appraisal as there are other important lessons to be learnt.

We next look at the Results (the R in SAARIE). The authors claim no difference between charity and paying patients, discuss a higher rate for surgeons in training (SIT) versus full time surgeons (FTS), and report a higher endophthalmitis for manual small incision surgery (MSICS). A closer look at their Tables 1and 2 tells a different story: “charity” is a confounding factor. Using charity cases as an exposure, the odds ratio for being a charity patient amongst those with endophthalmitis is 4.6, confidence interval (CI) 1.6 – 15. The authors feel the MSICS is the major culprit. But even if we examine MSICS alone, the odds of charity in endophthalmitis cases are 3.6 (CI 0.5 -26).

Their Table 1 shows us that FTS performed a total of 21284 MSICS.[2] As 2855 of these were full paying patients [Table 2], FTS performed MSICS on 18429 charity patients.[2] FTS had one endophthalmitis in the 2855 paying patients versus 21 in the 18429 charity cases. For MSICS performed by FTS, the odds ratio of being a charity patient amongst those with endophthalmitis was 3.4 (CI 0.5-25). The training level did not make too much of a difference: the incidence of endophthalmitis in MSICS operated on by FTS (21 of 18429) of 0.11% is not really different from that for MSICS operated on by surgeons in training (SIT) (12 in 8086) or 0.14%. Amongst those with endophthalmitis, the odds ratio for being operated on by an SIT was 1.7 (CI 0.9-3.2). The probability of endophthalmitis is clearly clinically significantly higher amongst the charity patients.

The Interpretation (I in SAARIE) of the study results requires insight into the cataract surgical scene in India. MSICS (versus phaco) and operations by trainee surgeons (versus FTS) are proposed risk factors for endophthalmitis, but the confounder of “charity”, or something associated with it is the true risk factor.

The data from the study strongly suggest that there is a difference in the treatment of paying and charity cases.

That pre- and postoperative examination as well as surgical protocols differ between paying and “charity” cases is no secret to any Indian ophthalmologist; some hospitals even have a policy of separate outpatient and operating rooms (ORs) for charity patients. It does not take a formal Bayesian analysis to combine such prior knowledge with the data to determine what is going on.

The authors claim a low endophthalmitis rate despite operating on patients from the lower socioeconomic strata and despite a large number of trainee surgeons. Poor hygiene, and, in an earlier publication the presence of *nocardia* in the soil have been provided as explanation for some of the cases.[6] If poor hygiene alone was the major factor, the endophthalmitis rate would be higher and other hospitals would report similar organisms as common pathogens. Under the circumstances we have to consider the alternative explanation that the data point to: the problem may lie in the protocols, especially the ones used for charity patients.

SIT are also considered part of the problem. However, if SIT are properly supervised and the protocols are followed, outcomes, especially endophthalmitis, should be no different from FTS. That has to be the basis of using humans for safe training.[7] The common factor seems to be the approach and possible breakdown of protocols in ‘charity’ patients.

It looks like the results cannot really be Extrapolated (E in SAARIE) to any setting. The basic premise of the shortcut protocols too is dubious: the authors feel that stringent sterilization and OR protocols were devised with other specialties in mind and do not have relevance to ophthalmology. Is a lens implant to be treated with less rigor than a bone marrow transplant or a hip replacement? And even if the fatal flaws discussed were not present, the data do not come anywhere close to suggesting that existing rigorous protocols be abandoned.

Use of the SAARIE also allows us to make suggestions for improvement and further study. The Aravind Eye Care System comprises high-volume hospitals that together can provide crucial information to change ophthalmic surgical protocols worldwide. I would urge them to consider an appropriately designed prospective study that addresses the limitations raised. And considering the enormity of the proposed change and the required “buy in”, I would also suggest that any such study utilize external input into planning and consider external validation of outcomes. Their quality assurance process could take cognizance of the data and try to eliminate the causes that make “charity” a risk.

Ophthalmic information is increasing exponentially and it is easy to be misled; critical appraisal skills provide a defense to some of the ill effects of information overload. I hope I have made a case for developing critical appraisal skills; I sincerely believe it should be made an integral part of our residency and fellowship training process.
